# Management of Pseudophakic Malignant Glaucoma in Sunset Syndrome: A Case Report and Literature Review

**Published:** 2019

**Authors:** Ioannis-Nikolaos CHALKIAS, Efthymios CHALKIAS, Anastasios HALKIAS

**Affiliations:** 1 Department of Ophthalmology, Xanthi General Hospital, East Macedonia and Thrace, Greece; 2 Department of Ophthalmology, AHEPA University Hospital, Thessaloniki, Greece; 3 Department of Ophthalmology, St. Lucas Hospital, Thessaloniki, Greece

**Keywords:** Sunset Syndrome, Monocular diplopia, Malignant Glaucoma, YAG-laser hyaloidotomy

## Abstract

To present an interesting case of pseudophakic malignant glaucoma in sunset syndrome, which potentially points to a correlation between a posterior chamber intraocular lens (PCIOL) subluxation and development of aqueous misdirection. Furthermore, we underlined the effectiveness of YAG-laser hyaloidotomy as a first line treatment for malignant glaucoma. This is a case report and literature review. A 76-year-old male with primary open angle glaucoma (POAG) with a history of left monocular diplopia due to inferior dislocation of the PCIOL (sunset syndrome), presented with a sudden onset of pain in the left eye and decreased visual acuity with corrected distance visual acuity (CDVA) of 20/60, seven years after an uneventful left phacoemulsification. The anterior chamber (AC) was shallow, the intraocular pressure (IOP) elevated and the PCIOL extruded in AC. Aqueous misdirection was diagnosed and treated with YAG laser hyaloidotomy. A gush of fluid emerged with simultaneous deepening of the AC and the IOP dropped immediately to 24 mmHg and later to 9 mmHg. A prophylactic YAG peripheral iridotomy was also performed. An IOL exchange surgery with anterior chamber IOL placement was performed a few days later resulting in a final CDVA of 20/30. We argue that postoperative subluxation of a PC IOL is likely to be an initiating event for aqueous misdirection.

## INTRODUCTION

Von Graefe described malignant glaucoma as a form of secondary glaucoma, marked by entrapment of the aqueous humor in the posterior chamber [1]. It is a rare complication that typically follows trabeculectomy surgery with peripheral iridectomy in patients with angle closure glaucoma [2], nevertheless it may also occur following cataract surgery [2] and laser iridotomy [3]. Aqueous misdirection is characterized by forward displacement of the lens-iris complex, a shallow central and peripheral anterior chamber (AC) and elevation of the intraocular pressure (IOP) [4]. Although, a number of different mechanisms leading to malignant glaucoma have been proposed, the precise pathology still remains unclear. A vicious cycle starts when the aqueous humor is trapped behind the vitreous, pushing the lens-iris diaphragm forward and narrowing the iridocorneal angle [4]. The triggering factors are believed to be a combination of anatomical characteristics of the eye (axial hyperopia), a narrow angle and inflammation of the ciliary body [5, 6]. We presented a unique case of aqueous misdirection in a patient with underlying sunset syndrome and proposed a new possible triggering mechanism of malignant glaucoma. 

## CASE REPORT

**Figure 1:**
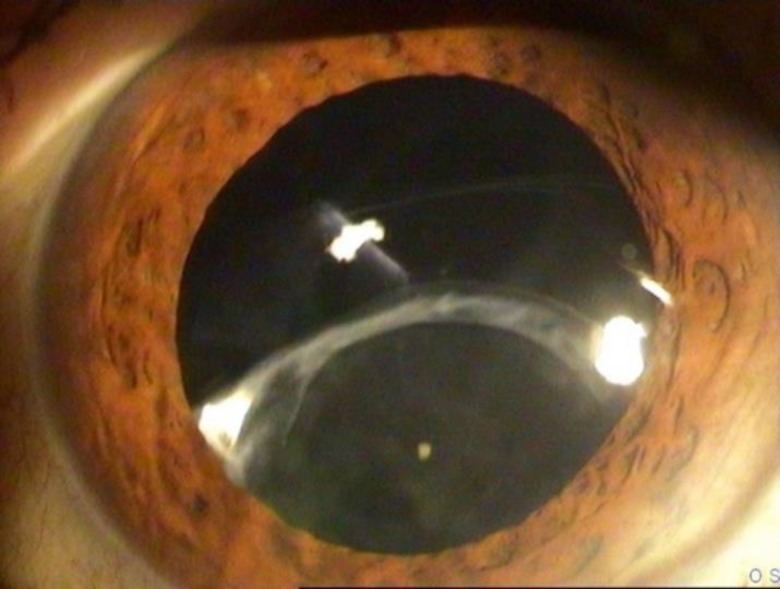
Left Eye Sunset Syndrome Evident as Downward Subluxation of posterior Chamber Intraocular Lens (PCIOL).

**Figure 2:**
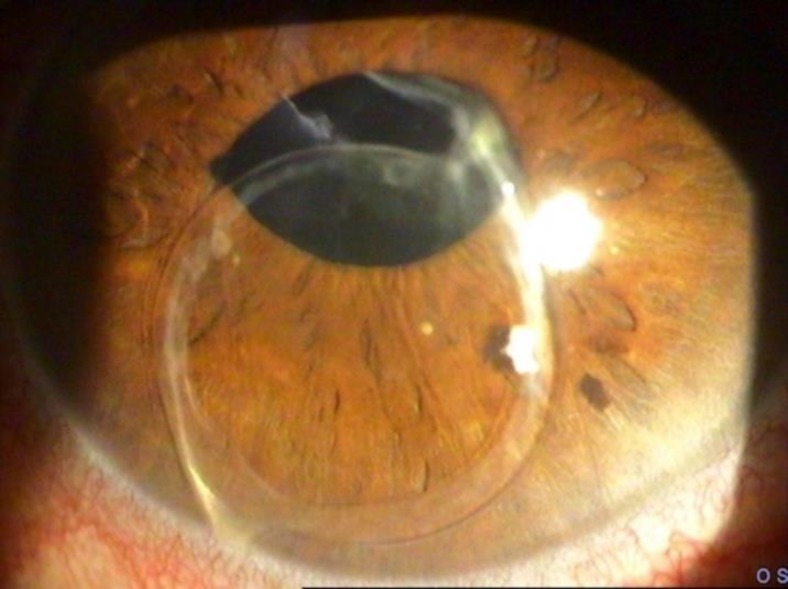
Dislocation of Posterior Chamber Intraocular Lens (PCIOL) Capsular Bag Complex to the Anterior Chamber (AC) of the Left Eye.

**Figure 3:**
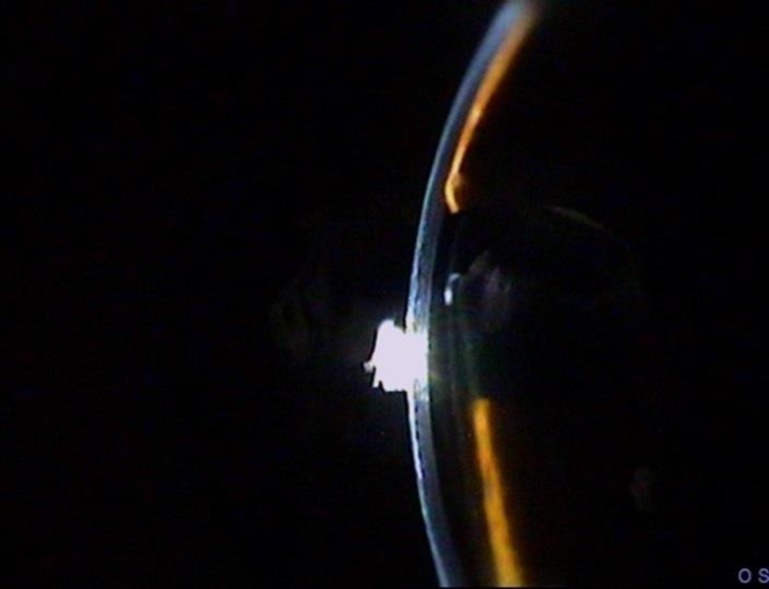
Flat Anterior Chamber (AC) in the Left Eye.

The patient was a 76-year-old male with an ophthalmic history of bilateral primary open angle glaucoma (POAG), who was under treatment since 2004 with no signs of pseudoexfoliation in either eye as confirmed by dilated pupil examination. Also, he had bilateral map-dot-fingerprint corneal dystrophy, bilateral tilted discs and bilateral mild dry age-related macular degeneration. He had a right phacoemulsification surgery in 2008 and an uneventful left phacoemulsification surgery with toric posterior chamber intraocular lens (PCIOL) implant in 2009. A written informed consent was received from patient. The study was conducted in accordance with the Declaration of Helsinki. In March 2016, he presented with left monocular diplopia due to inferior dislocation of the PC IOL (sunset syndrome-Fig 1). An IOL exchange surgery was scheduled, but while waiting to recover from an urgent abdominal aorta aneurysm repair surgery, he developed pain in the left eye and blurred vision. His corrected distance visual acuity (CDVA) was 20/60. Slit-lamp examination revealed displacement of the PCIOL - capsular bag complex to the AC (Fig 2), a very shallow - almost flat AC (Fig 3) and a rise in the IOP (30mmHg). The fundoscopic examination was unremarkable with no signs of choroidal detachment.

**Figure 4:**
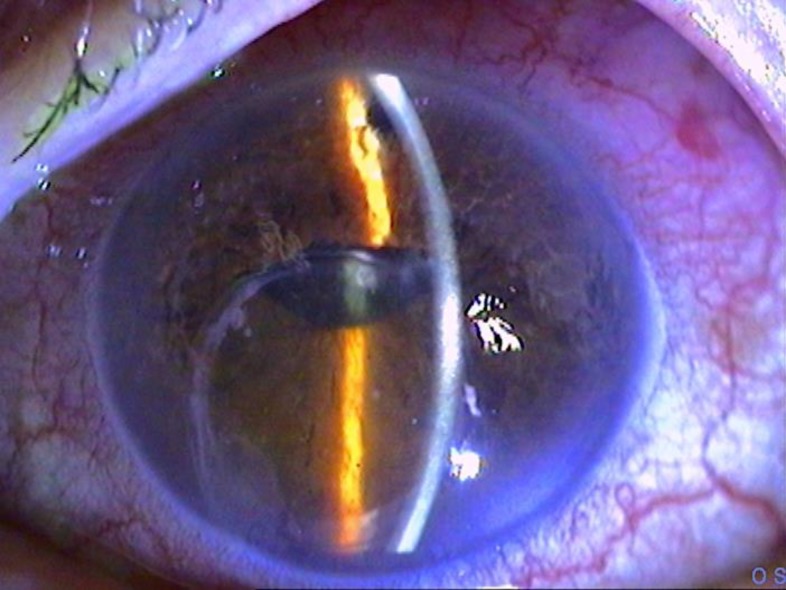
Deep Anterior Chamber (AC) in the Left Eye After YAG Laser Application to Disrupt the Hyaloid Face Just Above the IOL Rim, With Some Deeper Shots to Cut Across the Vitreous Humor. A Prophylactic YAG Peripheral Iridotomy Is Evident at 12.00 O'clock.

At this point, malignant glaucoma was diagnosed. YAG laser was used to disrupt the hyaloid face just above the IOL rim, with some deeper shots to cut across the vitreous humor. A gush of fluid emerged with simultaneous deepening of the AC (Fig 4) and the IOP dropped immediately to 24 mmHg and later to 9 mmHg. A prophylactic YAG peripheral iridotomy was also performed.

An IOL exchange surgery with anterior chamber IOL placement was performed a few days later resulting in a final CDVA of 20/30.

## DISCUSSION

Sunset syndrome has not so far been reported as the initiating event of the aqueous misdirection cascade. In our case, it could be hypothesized that the zonular brake caused a backward rotation of the ciliary processes (cilio-hyaloidal apposition) triggering the aqueous misdirection. 

Spontaneous luxation of an IOL with the capsular bag after an uneventful cataract surgery has been described before [7]. High myopia, pseudoexfoliation, diabetes mellitus, retinitis pigmentosa, pars planitis, myotonic dystrophy and increased age have been reported as predisposing factors [8-10].

In our case even though the patient did not show the ophthalmic characteristics of pseudoexfoliation (which would explain the zonular weakness), he had abdominal aortic aneurysm, which has a recognized association with this entity. Could he have a forme fruste pseudoexfoliation? Although high-resolution ultrasound biomicroscopy has been shown to be useful for detecting early deposits of pseudoexfoliation material on zonules [11] and help in the diagnosis of masked pseudoexfoliation syndrome, this examination was not performed, as it was not available.

Alternatively, the required intraoperative rotation of the toric IOL might have disrupted the integrity of the zonules resulting in delayed subluxation. This is the first time that a PCIOL was found displaced in the AC as a consequence of vitreous intumescence in the context of malignant glaucoma. Our case is also unique since the cascade that led to aqueous misdirection was initiated by the sunset syndrome and not by the initial phacoemulsification surgery per se which had taken place years earlier.

Medical management of malignant glaucoma includes the combination of cycloplegics, oral carbonic anhydrase inhibitors, topical β-blockers, apraclonidine and oral glycerol or intravenous mannitol, when needed [12]. However, only fifty-percent of patients would respond to medical treatment within five days [13]. Surgical options include transcorneal needling through a peripheral iridectomy [14], removal of the lens, posterior sclerotomy and vitrectomy [15].

In 2012, Pasaoglu et al. [16] described two patients with pseudophakic malignant glaucoma who were successfully managed by peripheral iridectomy, lens capsulectomy, hyaloidectomy and anterior vitrectomy using a vitreous cutter and thus demonstrated that aqueous misdirection can easily be managed by an anterior-segment surgeon. 

In a retrospective study conducted by Tang et al. [17], a number of different surgical techniques for the management of malignant glaucoma and their outcomes were studied and the authors found no significant differences between them. However, Debrouwere et al. [18] assessed different medical and surgical treatment options for malignant glaucoma and concluded that complete vitrectomy with iridectomy and zonulectomy (and phaco, if applicable) is the most successful method. 

Harbour et al. [19] clearly showed that pars plana vitrectomy (PPV) is an effective treatment option in both phakic and pseudophakic aqueous misdirection when other treatments fail. Krėpštė et al. [20] also supported this evidence and mentioned that after PPV the patient can discontinue his or her topical treatment.

In a pseudophakic eye, YAG-laser hyaloidotomy is a non-invasive method that can instantly disrupt the hyaloid face and lower the IOP absolving the patient from prolonged medical treatment with uncertain success or an unnecessary, more invasive procedure [21].

Irido-zonulo-hyaloido-vitrectomy (called mini-vitrectomy) is an effective and safe surgical procedure, similar to the one by Pasaoglu et al. [16] that has been described by Pakravan et al. [23] and provides a good alternative to PPV in the management of pseudophakic aqueous misdirection, where medical treatment and YAG-laser hyaloidotomy have failed. However, in their case the patient had a long-standing peripheral iridocorneal apposition (despite medication and a patent iridotomy) resulting in extensive peripheral anterior synechiae (PAS) and thus YAG-laser could not lower the IOP.

Our patient had a good control of his IOP with no PAS and this explains the effectiveness of YAG-laser. In 1996, Park et al. [22] published a case of aqueous misdirection in a pseudophakic patient where YAG-laser hyaloidectomy was unsuccessful and had to be treated with trabeculectomy and PPV.

## CONCLUSIONS

The posterior chamber intraocular lens inferior subluxation (sunset syndrome) can possibly act as a triggering mechanism to aqueous misdirection and YAG-laser hyaloidotomy is proven to be an effective way to instantly lower the IOP in an otherwise uncomplicated pseudophakic eye. 
